# Human dimensions of wildfires in NW Spain: causes, value of the burned vegetation and administrative measures

**DOI:** 10.7717/peerj.5657

**Published:** 2018-09-26

**Authors:** María Calviño-Cancela, Nuria Cañizo-Novelle

**Affiliations:** Department of Ecology and Animal Biology, University of Vigo, Vigo, Spain

**Keywords:** Forest fires, Survey, Public attitudes, Public opinion, Wildland-urban interfaces, Spain

## Abstract

Exploring the human dimensions of forest fires is a crucial, although often overlooked, aspect of wildfire research, since wildfires often have important socio-economic impacts and humans are nowadays the main cause of wildfires in many areas of the world. We carried out a telephone survey (*N* = 345 interviews) in one of the most fire-prone areas in Europe (NW Spain) in order to assess citizens’ awareness about wildfire causes and risks, their perception of the value of the vegetation and of administrative measures to fight against fires. Perceptions of respondents about fire causes were in general realistic although fires caused by pyromaniacs and for profit were overestimated, while vegetation management was comparatively underestimated. Citizens were broadly aware of the fire risk associated with different vegetation types, rightly considering native oak forests and agricultural fields as less risky than shrublands and pine and eucalypt plantations. Tree-dominated vegetation was more valued than treeless formations, and native forests more than tree plantations, which seems related to a preference for ecological value over utilitarian considerations. In addition, the value of eucalypt plantations was clearly affected by the education level of respondents, being less valued as the education level increased. Most citizens considered that the administration was not doing enough to fight against fires. The law that compels landowners to reduce fuels in wildland-urban interfaces was considered effective by most respondents (72%), but 50% considered it difficult to implement by landowners. This may explain the poor degree of compliance of this law.

## Introduction

Fire is a natural agent that has shaped natural landscapes and species adaptations over millions of years and has a significant role in the Earth system (Pausas & Keeley, 2009; Bowman et al., 2009). However, fire regimes have changed greatly due to anthropogenic causes, with an increase in the frequency of severe fires (Westerling et al., 2006; Marlon et al., 2008). Humans have a great influence on fire activity both directly, by causing, controlling and suppressing fires, and indirectly, by modifying the flammability of landscapes and warming the climate through the combustion of fossil fuels (Bowman et al., 2011). Wildfires may cause significant impacts, environmental as well as socio-economic (Pausas et al., 2008; Bowman et al., 2011), and this has motivated an increase in the interest of studying fire causes and risks. Addressing the causes of wildfires is essential for reducing them and, since fire causes are nowadays mainly anthropogenic in most areas of the world (FAO, 2007), it is crucial to explore the human dimensions of wildfires (McCaffrey et al., 2013).

Assessing public opinions and attitudes has become an important tool to support management decisions that are acceptable to the public, and to develop communication and environmental education strategies (Endter-Wada et al., 1998; Bennett et al., 2017). It is also important to understand people’s knowledge about wildfire risks, since a lack of awareness can lead to underestimated risks, to poorly-based decisions to prevent fires, or to less support for preventive measures (Winter & Fried, 2000). The public often perceives risks as more or less than what is reported in government statistics or research papers (Jacobson, Monroe & Marynowski, 2001). The study of human perceptions and awareness of fire causes, their consequences and governance policies is key to improving the effectiveness of management practices and information campaigns, as well as to increasing the legitimacy of governance processes, since it contributes to produce initiatives that are better matched to local contexts and are more socially acceptable (Lockwood et al., 2010; Bennett et al., 2017).

People’s views regarding the environment depend partly on characteristics of the individual and its context. Characteristics such as age, gender, education, and place of living constitute well-studied social bases of environmental concern and environmental risk perceptions (Vanliere & Dunlap, 1980; Finucane et al., 2000). When differences between segments of the public exists, this can help managers to predict when and from whom conflicts may arise when implementing plans or imposing rules. It may also help to identify to which segments of the population information campaigns should be addressed, or whether message differentiation among target segments is advisable (Bright, Newman & Carroll, 2007). For instance, previous studies have shown that men, older people, and people with higher levels of education tend to judge risks lower than women, younger people and people with lower educational levels (Savage, 1993; Finucane et al., 2000). Several studies also suggest that residence near forested areas and engagement in outdoor recreation are associated with increasing fire knowledge (e.g., Manfredo et al., 1990). Higher educational levels have also been shown to be related to higher understanding of local environmental issues, including wildfire issues (Cheng & Daniels, 2003; Depoe, Delicath & Elsenbeer, 2004; Gordon et al., 2010; Diaz, Steelman & Nowell, 2016), as well as to a higher acceptance of fire management strategies (Diaz, Steelman & Nowell, 2016).

In the study area (NW of Spain), as well as in other areas of the world (FAO, 2007), some of the main challenges of fire management policies are the increasing vulnerability of human settlements caused by the expansion of the so-called wildland-urban interface (areas where urban development meet or intermingle with wildland, where fires are frequent and the danger to human lives and properties can be higher; (Cohen, 2000; Calviño-Cancela et al., 2016; Calviño-Cancela et al., 2017), the increase in flammable biomass associated with the abandonment of land uses such as farming and ranching, and the expansion of highly flammable tree plantations (e.g., eucalypts and pines) (Lopez-Poma, Orr & Bautista, 2014; Viedma, Moity & Moreno, 2015). In fact, the type of vegetation has a very important role in determining the risk of wildfires (e.g., Calviño-Cancela et al., 2016; Calviño-Cancela et al., 2017; Bajocco & Ricotta, 2008; Carmo et al., 2011). Plant species may differ in flammability, for instance due to the presence of flammable volatile compounds in their tissues (Dimitrakopoulos & Papaioannou, 2001), and plant communities may differ in fuel loads or fuel continuity (Saura-Mas et al., 2010) or in the amount of light and heat reaching the ground, which influences the temperature and moisture content of fuels. In addition, in areas where fire causes are mainly anthropogenic (either deliberate, negligent or accidental), social factors, such as conflicts associated with the multiple uses of forests, urbanisation pressure, land abandonment or unemployment, become key drivers of the patterns of fire risk (e.g., Cardille & Ventura, 2001; Sebastián-López et al., 2008; Chas-Amil et al., 2015), which explains the differences in fire proneness between areas within and outside the wildland-urban interface for the same types of vegetation (Calviño-Cancela et al., 2016; Calviño-Cancela et al., 2017). A good understanding of these risk patterns is essential not only for policy makers, in order to design wildfire risk mitigation programs, but also for the general population, in order to implement wise prevention measures. In addition, the perceived value of different vegetation types by the public may modulate some behaviours related to the risk of fire ignitions. For instance, vegetation types less valued by people can be more vulnerable due to a lack of care, which can increase the risk of negligent fires (Barreal, Loureiro & Picos, 2012).

One of the most common approaches to manage fire risks focuses on fuel reduction activities, especially in wildland–urban interfaces, where the risk of fire is higher (Winter, Vogt & Fried, 2002). When this involves biomass management on privately owned land, it requires the active involvement of landowners, which may engage in those activities either voluntarily, for their own protection or the protection of lives, properties or natural resources they value, or coerced by law. Understanding the perceptions of the public in relation to the role of the administration in reducing fire risks is important for the effective implementation of fire fighting measures. Citizens are often poorly informed about government policies in relation to wildfires, and tend to be suspicious of the administration, particularly in rural areas, for policies often translate into restrictions regarding activities that are usual practice (Fiallo & Jacobson, 1995). A negative perception about the role of the administration can lead to non-collaborative behaviours and unwillingness to obey rules and recommendations (Tyler, 2006 [1990]; Stern, 2008).

Our goal in this study is to assess public awareness and attitudes regarding wildfires. We focused on wildfire causes, the risk associated with the type of vegetation, the value of this vegetation and the management measures implemented by the administrative authorities. More specifically, we analyse public views in relation to the relative importance of wildfire causes, including general causes, classified as deliberate, negligent, accidental or natural, as well as to more specific causes related to human behaviours and activities. Since the type of vegetation is one of the main factors determining fire proneness in a given area and, in contrast to other important factors such as topography or weather, it is subject to active management, we analyse in this study people’s views on the relative wildfire risk associated to the most common vegetation types in the region, as well as the perceived value of these vegetation types for the people. Since the administration has a prevalent role on fire management, we were also interested in determining public views about the role of the administration. Specifically, we analysed whether people considered that the administration was doing enough in the fight against fire, people’s view about the importance of implementing different possible improvements of fire fighting measures, and their views on a management measure designed to mitigate fire risk in the wildland-urban interface, since public views can determine the success or failure of administrative measures. We analyse general trends as well as differences across subgroups of the population, according to age, gender, educational level, residence in rural or urban areas and engagement in activities in the wild. We determine the degree of realism of public views by comparing respondents’ perceptions with real data from official reports, when available (for fire causes and the fire risk associated with different types of vegetation). As far as we know, this is the first study analysing the realism of public views in this regard.

The study was conducted in NW Spain, which has one of the highest incidence of wildfires in Europe (European Commission, 2016). This exceptionally high incidence of fires, mostly deliberate, together with the high population dispersion in the region and the large extension of the wildland-urban interface (Chas-Amil, Touza & Prestemon, 2010; Chas-Amil, Touza & García-Martínez, 2013) makes the analysis of the social aspects in relation with fire risks especially relevant. To our knowledge, this is the first study analysing public views in regard to wildfire risks and specific administrative measures for fire management in this fire-prone region.

## Methods

The study was carried out in Galicia (NW of Spain) which, similar to other regions of Southern Europe, has a very high incidence of wildfires. In the last decade (period 2005–2014), Spain had ca. 22% of all wildfires recorded in Europe and 30% of the burned area, and the NW is the region of Spain with the highest fire incidence (European Commission, 2016). In fact, Galicia represents only 6% of the Spanish territory but has 40% of the wildfires (MAGRAMA, 2012; period 2001–2010). There was an average of c. 4000 wildfires per year in the last decade, that burned more than 20.000 ha on average per year, which represents more than 0.75% of the Galician territory every year. Although estimations of the economic costs of wildfires are scarce, a study in 2014 estimated an average annual cost of fire extinguishment per hectare ranging between 5.3 € and 6.4 € (Vázquez Vázquez, Chas Amil & Touza, 2014). Moreover, most fires have human-related causes (99%), and most are deliberately lit (75%) (Chas-Amil, Touza & Prestemon, 2010), of which most are caused by farmers for management of the vegetation (e.g., bush clearing; 41%), followed by unspecified causes (other deliberate causes, not specified in official reports: 36%), with other causes such as those related to mentally ill people, ranchers for pasture regeneration or vandalism have frequencies lower than 10% (as estimated from official reports, period 2006–2011). The regional government of Galicia is the main competent authority in regard to forest fire management and has the task to elaborate every year a plan of prevention and defence against wildfires in Galicia (PLADIGA), which reflects the policy and the measures in relation to wildfire prevention, protection, surveillance, detection, extinction, research and development, information campaigns and staff training, including a detailed account of staff, budget and resources. The main goal of these yearly plans are to reduce, as far as possible taking into account the resources available, the ecological, economic and social consequences of wildfires, establishing specific goals every year in terms of the number and size of fires (PLADIGA, 2014–2017).

Galician population is very disperse (population density 92 people per km^2^ in 2016, with 2,718,525 inhabitants in an area of 29,575 km^2^; INE–National Institute of Statistics) which increases the contact between the people and the wildland in the so called wildland-urban interfaces, which represent 8% of the total area (for a detailed map of the distribution of wildland-urban interfaces in Galicia see Chas-Amil, Touza & García-Martínez, 2013). Most of the forest land in Galicia is privately owned (97%; PLADIGA, 2014–2017). Depopulation and farming abandonment in the last decades has led to an increase of forested land, especially of eucalypt plantations (Marey Perez, Rodriguez Vicente & Crecente Maseda, 2006), with relatively high risk of fire (Calviño-Cancela et al., 2016; Calviño-Cancela et al., 2017; Arellano et al., 2017).

For this study we undertook a telephone survey (*n* = 345 interviews) using randomly selected phone numbers from the White Pages directory, selecting respondents older than 18 yrs old (population = 2,344,376; error margin of 5% at 95% confidence). We made a total of 1,449 phone calls and the response rate was 24%. The survey was carried out in February-May 2016 and consisted of eight questions organized in three thematic parts (see Appendix A). In the first part we asked about the causes of fires, both general causes and more specific causes and motivations. The second part focused on the risk of fire associated with different types of vegetation and the value attributed to those vegetation types by the respondents. Lastly, the third part focused on the perceptions regarding the role of the Administration, the measures considered more important in the fight against fire and the effectiveness of the law that makes fuel reduction compulsory in the wildland urban interface. Fuel reduction is performed mainly by mechanical clearing or thinning of the vegetation. These three general topics constitute the main issues in relation to fires: the ignition causes, the vegetation as the key factor for fire propagation, and the role of the authorities with competence to implement management measures. In addition, we used official reports or previous studies in regard to causes and fire risk of vegetation types in order to contrast public perceptions with actual data. For this, we used the wildfire reports obtained from the Rural Affairs Department of the Regional Government (Xunta de Galicia) and the Spanish Ministry of Agriculture, Food and Environment (MAGRAMA) from the period January 1, 2006 to December 31, 2011 for wildfires occurred in Galicia. Wildfire reports list general information including location, date, burned areas and causes and motivations. We used the original classification in wildfire reports for general causes: natural, negligences, accidents and deliberate, but excluding rekindles, i.e., fires that start again after being extinguished, and unknown causes. For the more specific causes and motivations, we grouped the causes in 12 categories referring to human activities or behaviours the fire ignition was related to: agriculture and vegetation management (which refers to agricultural burnings, such as stubble burning, verge maintenance, bush clearing, control of animals considered harmful for crops or livestock and fires related to beekeeping), ranching (for pasture regeneration), forestry works, hunting, recreation, waste management (rubbish burning), profit gaining, conflicts, pyromaniacs (as referring to mentally ill people), accidents and natural (lighting) (Question 2 in Appendix A; see also Calviño-Cancela et al., 2017). We removed categories that were undefined, as they may potentially incorporate many different causes, i.e., other negligence, other deliberate and unknown. In regard to the risk associated to different vegetation types, we compared public perceptions with the results of previous studies analysing wildfire risk associated with different vegetation types in the same region (Galicia), based on official reports of fire occurrences (see Calviño-Cancela et al., 2016; Calviño-Cancela et al., 2017). These studies used a total of 11 vegetation types and analyse the risk of fire ignition and spread associated to these types of vegetation within and outside the wildland-urban interface (Calviño-Cancela et al., 2016) and in relation to topographical variables (Calviño-Cancela et al., 2017). Since such degree of complexity would make it very difficult for survey respondents to answer our questions, we made it simpler by using only six categories, easily recognizable by the general public: agriculture areas, shrublands, native oak forests, eucalypt plantations, pine plantations and mixed forest formations. Shrublands in this region are highly diverse and are dominated by native species, mainly of the Fabaceae (mostly *Ulex* spp. and *Cytisus* spp.) and Ericacea (with *Erica* spp., *Calluna vulgaris* and *Daboecia cantabrica*) (see e.g., Basanta et al., 1989; Calviño-Cancela, 2013). The most common native oak forests in the region are dominated by *Quercus robur* and are rich in biodiversity (Calviño-Cancela, Rubido-Bará & Van Etten, 2012; Calviño-Cancela et al., 2013; Calviño-Cancela, 2013). Eucalypt plantations in this region are mainly of *Eucalyptus globulus* and, more rarely *E. nitens*, with other eucalypt species being uncommon. Pine plantations are mostly of *Pinus pinaster*, *P. radiata* and *P. sylvestris*. Mixed forest formations refer to formations with a mixture of species, usually pines, eucalypts and oaks, frequently as a consequence of low management or abandonment.

At the end of the survey we included demographic questions, regarding the age, gender, educational level, place of residence (whether in urban or rural areas) and activity (whether the respondent performed activities in nature or not; referring to activities in the natural environment, such as forests, river or mountains, but excluding activities in highly humanized environments). Respondents were classified according to age in three groups: young adults (between 18 and 40 yrs old), middle-aged adults (41–65 yrs old) and senior adults (over 65 yrs old). The educational level was classified in three groups, lower educational level (up to primary education, usually until 12 yrs of age), middle educational level (with secondary education completed, usually until 16 yrs of age), and higher educational level (with upper levels, corresponding to upper secondary education or tertiary education).

The questionnaire was carried out anonymously. No personal information of the respondents was recorded. The study was approved by the University of Vigo’s Research Ethics Committee (201800004201). Before starting the questionnaire, participants were informed that this was a study of the University of Vigo about wildfires in Galicia, that their participation was voluntary and anonymous, and that they could terminate the interview at any time. The interviews started once participants provided their verbal consent. The whole interview, including an introduction and the questionnaire took about 15 min on average.

We used ordinal logistic regression to analyse the answers to most questions (except for questions 5 and 7). We analysed whether there were differences between answer options (main effect; for instance differences in the score given to causes or types of vegetation), and the effects of the interactions between the answers and demographic groups, in order to analyse whether there were differences between groups in their answers to our questions. Questions 5 and 7 were analysed with Pearson’s chi-squared tests, using contingency tables to test the independence between two categorical variables in order to analyse whether there were significant differences between demographic groups in the frequency in which they selected the different options given in the question. We used IBM SPSS Statistics for Windows to perform these analyses.

## Results

### Composition of the sample

The percentage of women and men in the sample (57% of women and 43% of men) did not differ significantly from that in the population (${\chi }_{\mathrm{1d.f.}}^{2}=2.978$, *P* = 0.084). Respondents were between 18 and 92 yrs old, and there were significant differences between the proportion of age groups in our sample compared to the population (${\chi }_{\mathrm{2d.f.}}^{2}=29.087$, *P* < 0.001), with a lower percentage of young adults (18–45 yrs old; 21% in our sample vs. 34% in the population), and a higher percentage of both middle-aged (45–65 yrs old; 46% in our sample vs. 40% in the population) and senior adults (>65 yrs old; 33% in our sample vs. 25% in the population). Regarding educational level, 27% of respondents had lower educational level, 49% had a medium level and 24% had higher educational levels, percentages that did not differ significantly from those in the population (${\chi }_{\mathrm{2d.f.}}^{2}=1.891$, *P* < 0.389). Respondents living in urban areas reached 71% and 29% resided in rural areas, while the percentage of respondents doing some activities in nature was 48% vs. 52% of respondents doing no activities in nature.

### Social perception of the frequency of fire causes

**Figure 1:**
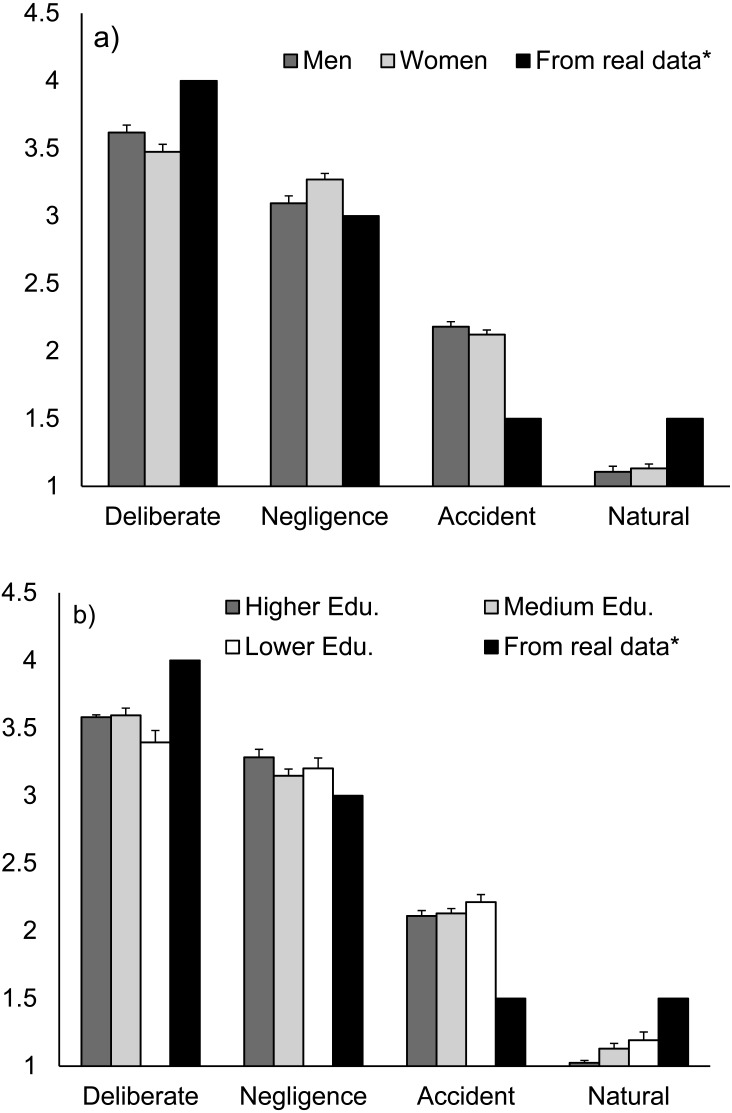
Respondents’ perception of the rank of general wildfire causes, for (A) men vs. women, and (B) different educational levels. Average ranking (±standard error) given by respondents, from 1 (the less frequent) to 4 (the most frequent). The rank of causes according to official fire reports (real data series) is also shown. Question 1 of the questionnaire (Appendix A).

Respondents perceived the general causes of wildfires (accidental, negligent, deliberated and natural causes) as being different in terms of frequency of fires caused by them. There were some differences (although small) between male and female participants and educational levels in the relative frequency they assign to different causes, as revealed by the significant interactions with these factors (Table 1), although the same general trend was observed for all demographic groups. Deliberate causes were considered as the most frequent fire causes, followed by negligent, accidental and, last, by natural causes, perceived as the less frequent (Fig. 1). Men assigned more frequency than women to deliberate causes and less to negligence (Fig. 1A). Differences between men and women were smaller in regard to the frequency of accidents and natural causes (Fig. 1A). Differences associated with the educational level were also small but there was a trend to assign more frequency to deliberate causes among people with medium and high educational levels, while the lower the educational level the higher the perceived frequency of accidental and natural causes (Fig. 1B). The perception of deliberate as the most frequent followed by negligent causes was realistic, in accordance with official fire reports (Fig. 1). Accidents and natural causes have similar frequencies according to official reports, but accidents were considered more often as more frequent than natural causes by respondents (Fig. 1).

**Table 1:** Results of the ordinal logistic regression analyses of citizens’ responses to questions 1 to 4 and 6 of the questionnaire (Appendix A). Results of the analyses are shown in different columns for the score given by respondents to general and specific wildfire causes according to the perception of their frequency (questions 1 and 2), to types of vegetation according to perceptions of their associated risk of wildfire and of their value (questions 3 and 4), and to administrative measures according to the importance of improving them in order to better suppress fires (question 6). See questionnaire in Appendix A.

	Chi^2^(likelihood ratio), d.f., *P*				
Source of variation	General causes (Q. 1)	Specific causes (Q. 2)	Vegetation (risk) (Q. 3)	Vegetation (value) (Q. 4)	Admin. Measures (Q. 6)
Answers	1756.34, 4, <0.001	1662.61, 10, <0.001	90.45, 5, <0.001	804.96, 5, <0.001	450.84, 7, <0.001
Age: Answers	14.63, 8, 0.067	27.30, 22, 0.200	19.92, 12, 0.069	13.63, 12, 0.325	19.92, 16, 0.224
Sex: Answers	14.30, 4, 0.006	18.35, 11, 0.074	14.39, 6, 0.026	2.69, 6, 0.846	8.46, 8, 0.390
Residence: Answers	5.05, 4, 0.283	17.64, 11, 0.090	1.53, 6, 0.957	32.86, 6, <0.001	7.39, 8, 0.496
Outdoor activities: Answers	6.06, 4, 0.195	11.23, 11, 0.425	15.70, 6, 0.015	6.38, 6, 0.382	12.21, 8, 0.142
Educational level: Answers	16.09, 4, 0.041	29.20, 22, 0.139	21.16, 12, 0.048	21.87,12, 0.039	52.02, 16, <0.001

**Figure 2:**
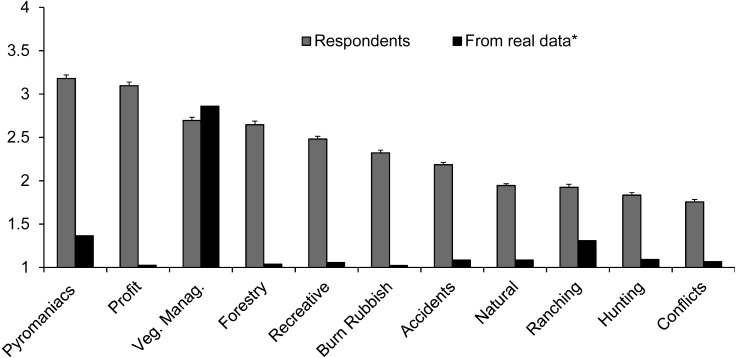
Frequency (mean ± standard error) of specific fire causes as perceived by respondents. Frequencies are shown in a scale from 1 (for a cause considered as infrequent) to 4 (very frequent). The frequency of specific fire causes according to official fire reports (real data series) is also shown, transformed to a scale from 1 (0% of fires) to 4 (100% of fires). Question 2 of the questionnaire (Appendix A).

When looking at the more specific fire causes (Question 2 in the questionnaire; Appendix A), again the population perceived them as differing in frequency, but with no differences between demographic groups in their perceptions (no significant interactions between causes and demographic groups; Table 1). Fires caused by pyromaniacs (mentally ill people) and those caused for profit were considered the most frequent, being clearly overestimated compared to official reports (Fig. 2). These were followed by fires caused for vegetation management, which were rightly considered as quite frequent, although underestimated with respect to fires caused by mentally ill people or for profit. The rest of causes, that followed in terms of perceived frequency, were all overestimated, as they cause a relatively small number of fires according to official reports, with ranching being the most frequent among them (Fig. 2). Ranching was perceived as similarly frequent as natural causes, hunting or conflicts, while it brings about ca. 3.5 to 4 times more fires than those causes (5.5% of fires compared to less than 1.6%).

### Social perception of the risk associated with different types of vegetation

**Figure 3:**
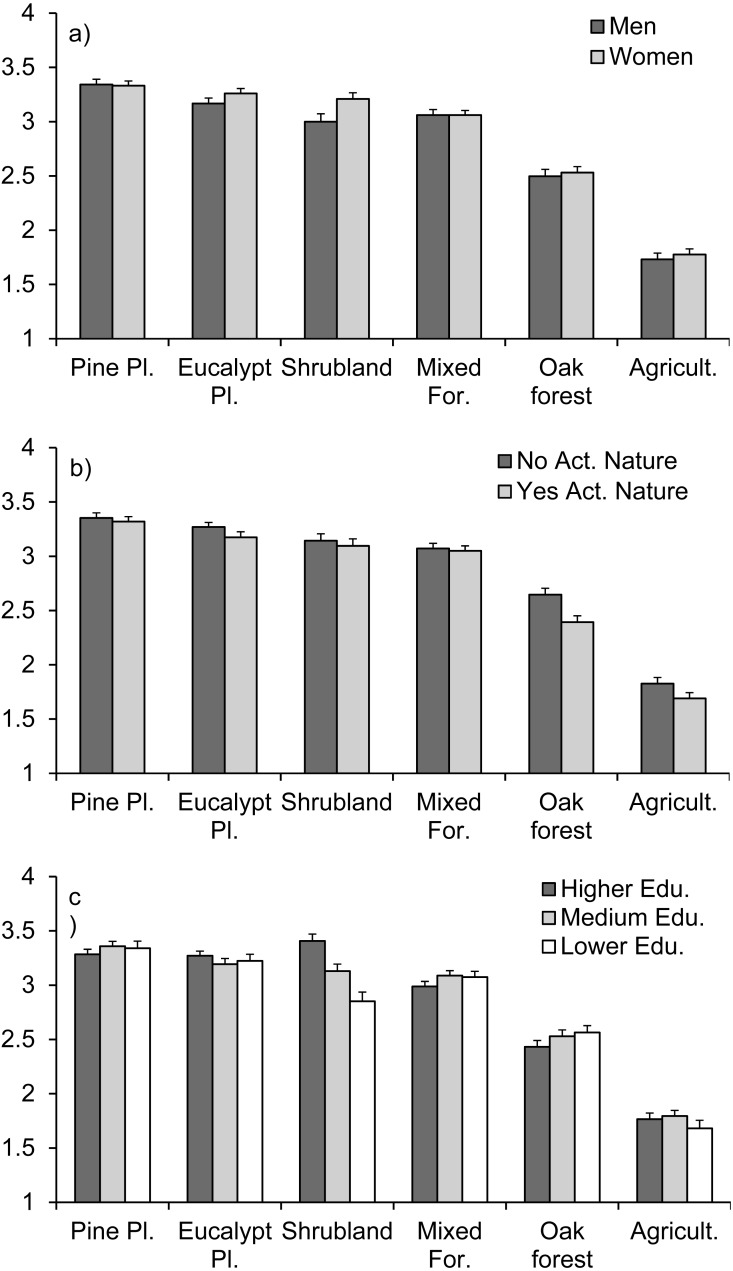
Respondents’ perception of the ignition risk of different types of vegetation according to (A) men vs. women, (B) people that perform activities in nature or not, and (C) different educational levels. The average ranking (±standard error) given by respondents to each vegetation type is shown, from 1 (lowest risk) to 4 (highest risk). Question 3 of the questionnaire (Appendix A).

There were significant differences between vegetation types in their risk of fire, as perceived by the population (Table 1). Overall, pine plantations, eucalypt plantations, shrublands and mixed forested areas were considered the most fire prone, while native oak forests and agricultural fields were considered as having lower risk of fire (Fig. 3), as is broadly confirmed by real data (Calviño-Cancela et al., 2016; Calviño-Cancela et al., 2017). There were differences between some demographic groups in their perception of the risks: between male and female participants, between people that performed activities in nature and people that do not, and depending on the level of education (significant interactions with these factors, although only marginally significant for the educational level; Table 1). Women perceived shrublands as relatively riskier compared to men, and the same with eucalypt plantations, although differences with men were smaller for this type of vegetation (Fig. 3A). People not performing activities in nature perceived all vegetation types as more risky than people performing activities in nature, with differences being more pronounced for native oak forests and, to a lower degree, agricultural fields and eucalypt plantations (Fig. 3B). In regard to the educational level, there was a perception of higher risk associated with shrublands as the educational level increased, while differences regarding other vegetation types were smaller (Fig. 3C).

### Social perception of the value of different types of vegetation

**Figure 4:**
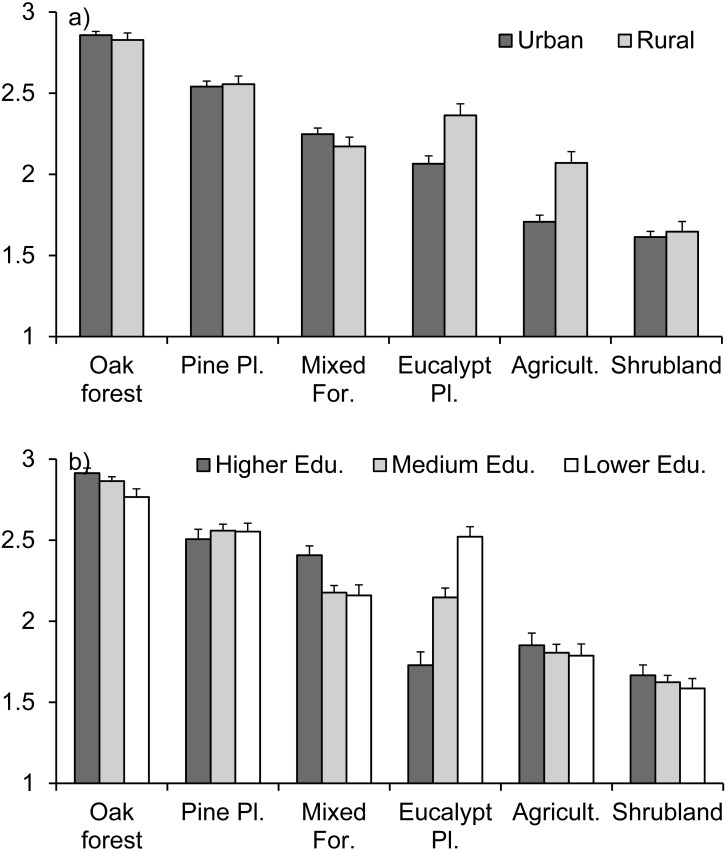
Respondents’ perception of the value of vegetation types, as estimated by the importance of the loss in case of destruction by fire, for (A) urban vs. rural areas and (B) different educational levels. Average ranking (±standard error) given by respondents to each vegetation type, with 1 indicating low importance of the loss, 2 indicating important loss, and 3 indicating very important loss. Question 4 of the questionnaire (Appendix A).

There were significant differences between vegetation types in regard to their value for respondents (Table 1), assessed as the importance of the loss in case of a fire. Overall, native oak forests were the most appreciated by the population, followed by pine plantations, mixed forested areas, eucalypt plantations, agricultural fields and, last, shrublands. But this pattern differed depending on the place of residence (urban vs. rural areas) and the educational level (significant interactions with these factors; Table 1). Respondents in rural areas gave more value to eucalypt plantations and agricultural fields than people in urban areas. In regard to educational levels, differences were especially noticeable for eucalypt plantations, which were more valued by respondents with lower educational level, recording a similar score to that of pine plantations, while respondents with higher educational levels gave eucalypt plantations the lower score together with shrublands (Fig.  4).

### Social perception of the role of the administration

**Figure 5:**
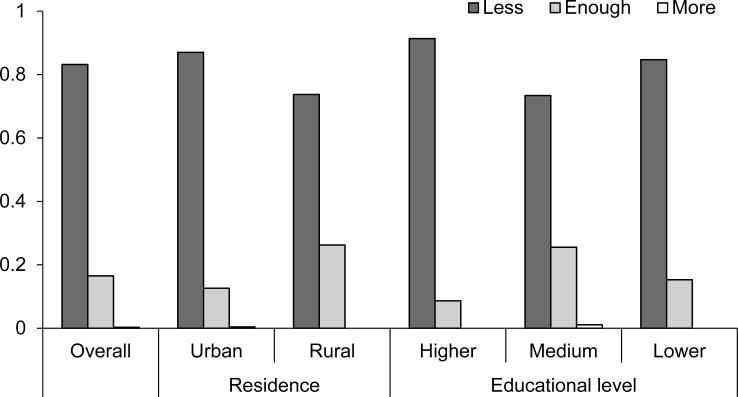
Respondents’ perception of the role of the administration in the fight against wildfires. Percentage of people considering that the administration was not doing enough, enough and more than enough, respectively, for the overall population, and depending on the place of residence and the educational level. Question 5 of the questionnaire (Appendix A).

Most people considered that the administration was doing less than enough in the fight against wildfires (83%; Question 5 of the questionnaire; Appendix A), while 17% considered that the administration was doing enough, and less than 1% considered that they were doing more than necessary (Fig. 5). There was a significant effect of the place of residence and of the educational level on this perception (Table 2), with the percentage of people considering that the administration was doing less than necessary being higher among people from rural areas than from urban areas, and also among people with medium educational level (Fig. 5).

**Table 2:** Results of the Pearson’s chi-squared test of the respondents’ perception on the role of the administration. Results of citizens’ responses to question 5, in which they were asked whether the administration was doing enough in the fight against fire, and to question 7, in which they were asked about the effectiveness and feasibility of the implementation by landowners of the law that makes fuel reduction compulsory in the wildland-urban interface. See questionnaire in Appendix A.

	Chi^2^ (Pearson), d.f., *P*	
Source of variation	Role of Admin. (Q. 5)	Fuel reduction (Q. 7)
Age	9.22, 4, 0.056	4.332, 4, 0.363
Sex	0.80, 2, 0.669	0.173, 2, 0.917
Residence	9.87, 2, 0.007	1.459, 2, 0.482
Outdoor activities	1.59, 2, 0.451	1.347, 2, 0.510
Educational level	12.27, 4, 0.015	7.010, 4, 0.135

**Figure 6:**
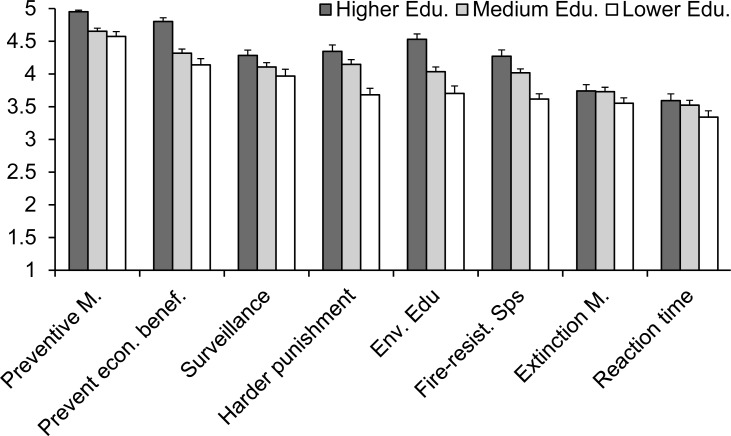
Respondents’ perception of the importance of improving different administrative measures to better suppress wildfires for people with different educational levels. The average score (±standard error) given to each measure is shown, from 1 (for measures whose improvement has the lowest priority) to 5 (for those with the highest priority). Question 6 of the questionnaire (Appendix A).

In regard to the importance of improving administration measures in the fight against wildfires (Question 6 of the questionnaire; Appendix A), all the proposed measures attained high scores (3.5 to 4.7 on average, in a scale from 1, lowest importance, to 5, highest), which means that the population considered important to improve all measures, but with differences between measures and a significant interaction with the educational level (Table 1). The improvement of preventive measures and a more restrictive legislation were the measures considered as the most important for respondents, while measures related with fire extinction (increasing the efforts in extinction and reducing the reaction times) were considered, in comparison, less important. Respondents with higher educational levels considered more important the improvement of all measures, but this trend was especially clear for legislative measures (for a more restrictive legislation and harder punishment), environmental education and plantation of fire-resistant species (Fig. 6).

The social perception about the effectiveness of the law that makes fuel reduction compulsory in the wildland urban interface was uniform among demographic groups (Table 2). About half of the respondents (51%) considered this an effective measure but difficult to implement by landowners, 28% of people considered it an ineffective measure and 21% considered it effective and feasible to implement by landowners.

## Discussion

Our sample was representative of the population in terms of gender proportion and educational levels, but slightly biased toward older ages. In any case, we introduced the demographic groups as factors in all the analyses, thus limiting the impact of biases.

### Social perception of the frequency of fire causes

Respondents demonstrated being aware of the importance of deliberate causes of wildfires in the region, rightly considering them as the most important causes of wildfires. Although the general trend was the same among demographic groups, it is interesting to note the increase in the perceived importance of causes in which humans have a clear responsibility (deliberate causes) among people with higher educational levels, in contrast with respondents with lower educational levels, which gave more importance than the more educated to natural causes and anthropogenic but involuntary causes (accidental). This might be related to a more questioning attitude of the more educated people regarding human responsibility for environmental problems (Díez-Nicolás, 2006).

In regard to more specific causes, the role of pyromaniacs (mentally ill people) was clearly overestimated, as was that of deliberate fires caused for profit. The incidence of fires caused by pyromaniacs is often overestimated, not just by the general population but even by fire reporters and officials, due to the poor understanding of this mental disorder, so that the term pyromaniac is often wrongly applied to people that set fires deliberately (arsonists in the strict sense), even when no mental illness has been diagnosed (Doley, 2003 and references therein). However, it is important to note that, in our questionnaire, we referred to pyromaniacs explicitly as mentally ill people, so that the overestimation does not come from an inappropriate use of the term but from a wrong perception of the real incidence of this cause. The terms arsonist and pyromaniac are regularly used interchangeably in the media, despite their very different meanings and implications, and this contributes to creating confusion in the population. Pyromaniacs engage in intentional and pathological firesetting, while arsonists wilfully and maliciously set fire (or aid in setting fire). Of those arrested for the crime of arson, ca. 10% are considered to be mentally ill (Barker, 1994). A good understanding of deliberate firesetting behaviours is important for improving strategies to control and manage fire ignitions. An important aspect in this regard is recidivism and associated risk factors. The rate of firesetting recidivism is relatively low compared with the rate of general redicivism related to other offences (more than 55% of firesetters have subsequent charges for any offence while only c. 5% have subsequent charges for firesetting; Ducat, McEwan & Ogloff, 2015). The highest probability of re-offending is in the first two years, and recidivist firesetters are likely to have mental disorders (Ducat, McEwan & Ogloff, 2015 and references therein).

The consideration of vegetation management as less important than pyromania and profit-driven fires was unrealistic, since it is, in fact, the most important specific cause in the study area (33% of fires; period 2006–2011). This was due to an overestimation of pyromania and of fires caused for profit rather than to an underestimation of the frequency of fires related to vegetation management. These fires correspond mostly to agricultural burnings for waste disposal. Their high frequency has led authorities to develop strict regulations in this regard, banning all agricultural burnings in summer and making it compulsory to ask for specific permits the rest of the year, so that rangers are aware and can supervise the burning. There is a lack of awareness in the population about vegetation management being a major cause of wildfires, which highlights the importance of information campaigns, since the effectiveness of preventive measures depends on the wise behaviour of thousands of landowners that dispose of their vegetal waste every year. The strategy followed by the administration to reduce the high incidence of vegetation management as a cause of wildfires has been based on imposing strict regulations. However, farmers still use fire as a management tool, and this causes a great number of fires every year. Reducing the risk of this routine practice is needed. To this end, education and training campaigns especially focused on this particular cause of wildfires should be promoted, in order to raise awareness of the danger involved as well as to show how to improve safety when using fire.

Ranching was rightly considered as relatively infrequent but somewhat underestimated in comparison with other causes such as natural causes, hunting or conflicts. Fires related to ranching occur mainly in shrublands (Calviño-Cancela et al., 2017), where fire is traditionally used in relation to extensive livestock grazing, to provide a flush of nutritious re-growth (Webb, 1998). The incidence of ranching was higher outside the wildland urban interface (ca. two times higher; Calviño-Cancela et al., 2017), thus far from populated areas, which may contribute to a relative underestimation in comparison with other causes. The rest of causes specified in the questionnaire have lower incidence in the study region (<2% each).

### Social perception of the risk associated with different types of vegetation

The realism of public perceptions about the fire risk associated with different vegetation types, considering agricultural fields and oak forests as the less fire prone, is in accordance with real data (Calviño-Cancela et al., 2016; Calviño-Cancela et al., 2017), which is noticeable. This is especially so in the case of oak forests since, despite their similarity to tree plantations in terms of structure, respondents clearly differentiated them in terms of fire risk. Of all the land covers considered in Calviño-Cancela et al. (2016), oak forests had the lowest ignition risk which, pooling together areas within and outside the wildland-urban interface, was 2.7 and 22 times lower than in “pure” eucalypt and pine plantations, respectively (considering pure plantations those with at least 70% dominance of eucalypts or pines, respectively). This difference is even higher when compared with mixed plantations, where eucalypts or pines are still dominant but have lower land covers. Oak native forests in the region, in contrast with pine and eucalypt plantations, are characterized by the deep shade provided by their canopies, which leads to lower temperature and higher moisture under the canopy, and limits the growth of biomass (Calviño-Cancela, Rubido-Bará & Van Etten, 2012), all contributing to reduce the risk of ignition. In addition, the high content in flammable volatile essential oils of pine and eucalypt leaves adds in flammability (Dimitrakopoulos & Papaioannou, 2001; Schwilk & Ackerly, 2001). Although oak forests were rightly considered less risky than tree plantations and shrublands, they were perceived as riskier than agricultural fields, when they are in fact ca. 4 times less risky (in the whole territory, pooling together the areas within and outside the wildland-urban interface; Calviño-Cancela et al., 2016). However, native forests are indeed riskier than agricultural fields in the wildland-urban interface (Calviño-Cancela et al., 2016), i.e., areas closer to most of the population, which likely contributes to a more widespread perception of the risk associated with this type of vegetation in the general population. Interestingly, people engaged in activities in nature considered all vegetation types less risky than people not performing those activities, which might be related to a higher tolerance of the risk by people obtaining a benefit from the recreational use of the vegetation (Slovic, Fischhoff & Lichtenstein, 1982).

### Social perception of the value of different types of vegetation

The higher perceived value of tree-dominated vegetation compared to treeless formations coincides with a previous study in the same area upon which vegetation with trees was perceived as more attractive than shrublands (for 96% of respondents; Lage-Picos, 2001). A low preference for treeless environments has been shown by a number of studies in Europe, the USA and Australia (e.g., Lamb & Purcell, 1990; Cook & Cable, 1995; Kiley et al., 2017). The preference for native forests over plantations in our study seems to be more related to the aesthetic or perceived ecological value of these formations rather than to their economic value, since plantations provide more revenue. In fact, the European public attaches higher value to forest conservation and protective functions than to forest utilisation aspects, and the preservation of biodiversity is perceived as one of the most important functions of forests, with no significant differences among regions (European Commission, 2009). This may reflect a societal trend towards a greater appreciation of natural values and a more critical attitude towards resource exploitation (European Commission, 2009). In addition, oaks, the dominant trees in native forests, are, together with chestnuts (also abundant in these forests) the most appreciated trees by the Galician public, while eucalypts are the least valued (Lage-Picos, 2001). This seems to be related to the native compared to alien status of species, with oaks and pines being considered native by most people (although some frequently planted pines such as *P. radiata* are non-native in this region) while eucalypts are recognized as alien species (Lage-Picos, 2001). It is noticeable the low value perceived of shrublands, which, in addition to their low economic profitability (used almost exclusively for extensive ranching nowadays) are usually perceived as having low natural value compared to tree-dominated communities (Lage-Picos, 2001). However, shrublands harbour a rich native biodiversity in this region, with many species of conservation concern (Calviño-Cancela, Rubido-Bará & Van Etten, 2012; Calviño-Cancela, 2013). Regarding agricultural fields, their perceived value increases in rural areas, where there are obviously more farmers, and therefore greater awareness of their economic importance, than in urban areas. Rural people often express higher preference for scenes of agriculturally modified landscapes than do urban people (Orland, 1988). Eucalypt plantations were also more valued in rural areas, which may also be related to their economic importance there. In a region where most forest land is in private hands, sales of eucalypt wood have a significant impact on household income in rural areas (Cátedra, 2015). But the most remarkable aspect in relation to the perceived value of eucalypt plantations is the pattern observed with educational level: the higher the educational level, the lower the valuation of eucalypt plantations. The value of eucalypt plantations was similar to that of pine plantations for people with lower educational level but was one of the least valued for higher-educated people. As commented, eucalypt plantations are a significant source of income in rural areas but have a very limited ecological value, harbouring less biodiversity than pine plantations, native oak forests or shrublands in the study region (Calviño-Cancela, Rubido-Bará & Van Etten, 2012; Calviño-Cancela et al., 2013, Calviño-Cancela, 2013). In addition, eucalypts are non-native in this area and have an invasive character (Sanz-Elorza, Dana & Sobrino, 2001; Richardson & Rejmanek, 2011), being able to spontaneously spread in native forests, plantations and shrublands (Calviño-Cancela & Rubido-Bará, 2013; Calviño-Cancela, Lorenzo & González, 2018). Its limited value from the point of view of biodiversity conservation, in addition to its non-native status and the environmental problems associated with non-native species (Pimentel et al., 2001) might explain its perceived low value for higher-educated respondents. The educational level tends to be positively correlated with environmental awareness (Liordos et al., 2017; Vaske, Jacobs & Sijtsma, 2011), with more educated people tending to give relatively more importance to environmental aspects over exploitation aspects (Teel & Manfredo, 2010).

The perceived value of vegetation types might affect their ignition risk, for the efforts each individual is willing to invest in preventing fires might be correlated to the value attributed to each type of vegetation. In fact, a previous study has found a negative relationship between the price of the land and the number of fires and the area burned, so that more expensive land is less affected by fires (Barreal, Loureiro & Picos, 2012). Social perceptions can also affect priorities in firefighting operations. For instance, shrublands are perceived as having low value and this may increase both the risk of ignition, very high in this land cover, and the size of fires, for firefighters often concentrate their efforts in more valued land covers (Moreira et al., 2009).

### Social perception of the role of the administration

The survey reveals a general poor perception of the role of the administration, which, according to most respondents, is not doing enough in the fight against fires. This negative perception increased among urbanites, which have a less direct contact with wildfires and administrative measures in the field, so that their negative perception may have been influenced by media filters (Jacobson, Monroe & Marynowski, 2001). The improvement of preventive measures, including legislative reforms and environmental education, was considered the most important, especially by respondents with higher education level, while the improvement of extinction measures was considered less critical, similarly for all levels of education. The trend in the number of fires and the area burned in the last four decades (1976–2015; PLADIGA, 2014–2017) gives support to this perception: the area burned per decade has followed a steady downward trend (of 25% to 35% per decade), despite an ascending trend in the number of fires in the three decades from 1976 to 2005 (of 85% to 120% per decade), which suggest that extinction measures are being more effective than measures to prevent new ignitions. In fact, prevention remains as the biggest challenge: only in the last decade has a decrease in the number of fires been finally achieved (of 65% compared to the previous decade; PLADIGA, 2014–2017), but it remains as one of the highest rates in Europe, with 0.13 fires per km^2^ on average, only exceeded by Portugal (European Commission, 2016). As recommended by FAO (2007), policymakers should move from a reactive attitude after the fire outbreak to a proactive approach of planning and prevention. This should translate to increasing budgets for prevention and decreasing funding for suppression, which should be less needed.

Among preventive measures, developing legislation to prevent people to get economic benefits from wildfires was especially favoured by respondents overall. This reflects a common perception of fires being still used by some for profit, with legislative action being perceived as an important measure to prevent fires. The need for education campaigns was especially favoured by people with higher educational level, who tended to be more critical with the role of the administration. Information campaigns can influence public acceptance of management strategies (Shelby & Speaker, 1990). However, wildfire prevention campaigns in the study region are usually targeted to the general public using mass media, are often superficial in their content and do not usually provide useful information to help individuals avoid risky behaviours. Approaches in which the public are just passive recipients of information without being involved in reciprocal action do not usually produce significant changes in perceptions or behaviours (Beringer, 2000). More constructive campaigns focused on education and active training to specific segments of the population are recommended, in particular to improve awareness of the risk involved in some activities, such as the use of fire for vegetation management in agriculture, and to provide information on how to improve safety when using fire. Awareness is essential since it increases motivation to engage in action to prevent wildfire risks and, without motivation, interventions to improve knowledge and training often have modest impacts on behaviours (Visser, Holbrook & Krosnick, 2007).

Preventive measures against wildfires are often focused on fuel reduction (e.g., in Spain; MAGRAMA, 2012). Fuel management is often controversial worldwide (Gill, Stephens & Cary, 2013). Lack of public support can result in failure to implement fuel management strategies adequately (Toman et al., 2011; Ryan, Knapp & Varner, 2013). Understanding the social acceptability of fuel management strategies is therefore of great importance for their success (Toman, Shindler & Brunson, 2006). Previous research on public attitudes toward fuel reduction techniques has focused primarily on prescribed burning (Manfredo et al., 1990; Jacobson, Monroe & Marynowski, 2001), which is very seldom used in the study region. In fact, in a study on practitioner perceptions of wildland fire management across South Europe and Latin America (Molina-Terren, Cardil & Kobziar, 2016), the North of Spain (including the study region) was the region with the lowest affinity for prescribed fire use: most respondents believed that prescribed fire did not have positive ecological effects and half thought that it was not socially acceptable, although most people were of the opinion that this practice would increase in the future. Fuel reduction in the study region is usually performed by mechanical thinning, which involves removing or altering the structure of trees or understorey to reduce the amount of combustible fuels, in order to reduce fire intensity and to change fire behaviour. Common arguments against this practice include its high cost, visual impact, reduction in available habitat for plant and animal communities, water quality and potential to favour weeds (Winter, Vogt & Fried, 2002; Brooks et al., 2006). In our study, although most respondents considered the law that compel landowners to reduce fuels in the wildland-urban interface as effective, half of respondents considered it difficult to implement. In fact, the degree of compliance of the law is low and the risk of ignition in these interfaces remains high (more than 50% higher within the wildland-urban interface than outside, (Calviño-Cancela et al., 2016). The NW of Spain has very good conditions for the growth of biomass (Riesco, 2004), thus fuel management requires a constant effort. The high cost of implementing this measure reduces its effectivity, for many landowners choose not to obey the law, despite the consequent risk of being punished. A less costly alternative could focus on the establishment of fire-resistant vegetation. In this regard, it is important to highlight the low fire risk of native oak forests, which have also high resistance and resilience to fire (Proença, Pereira & Vicente, 2010), and can establish naturally in most areas in this region (Izco, 1987), thus requiring minimal management efforts. The use of fire-resistant species as a management measure against fires was considered relatively important by the population, especially by the more educated, and oak forests were rightly perceived as having low fire risk and were highly valued by the people. Favouring the use of this type of vegetation as an alternative to fuel reduction, both within and outside the wildland-urban interface, would thus represent a socially acceptable measure with likely positive outcomes, both in terms of biodiversity conservation an economic sustainability. Increasing the economic sustainability in the fight against wildfires is crucial, since the previously commented positive outcomes in terms of reducing the area burned come at a very high cost. The annual spending of the Government in this regard goes as high as $50 of public money per ha of wildland (period 2014–2017, PLADIGA, 2014–2017). This is difficult to sustain in the long term, thus finding affordable fire management options becomes a priority. Integrated approaches based on fuel management that take advantage of the adaptations of the native vegetation can constitute effective alternatives that maximize the ecological benefits while minimizing socio-economic impacts (Hirsch et al., 2001; Fernandes, 2013).

## Conclusions

Our study, based on a telephone survey to the general public in the NW of Spain, one of the most fire-prone areas in Europe, revealed that the population has a quite realistic perception of general fire causes and the risk posed by wildfires in different vegetation types, and attributed more value to forested areas than to tree-less formations, and to native forests than to plantations. Nevertheless, people seems not fully aware of the risk involved in the use of fire for vegetation management, the human activity causing most fires in the area, and overestimate the role of mentally ill people (thus not responsible of their acts) and the importance of fires motivated by profit gaining. Our results highlight the importance of the educational level in affecting citizens’ views; more educated people was more critical with the role of the administration and attributed less value to eucalypt plantations, an economically important but controversial land use due to its non-native status in the area. In line with citizens’ preferences, a more proactive approach in the fight against fire is recommended, focusing more on prevention than in suppression. Among preventive measures, those focusing on changing behaviours of individuals are especially recommended, since most fires are caused by humans in this region. Information and training campaigns, which have been especially favoured by people with higher educational level, can be especially useful in this regard. More constructive and focused campaigns targeted at specific segments of the population are recommended, in particular to improve awareness of the risk and increase the safety of some particularly hazardous activities. Among them, the use of fire for vegetation management in agriculture requires the most attention, since it is a major cause of wildfires every year. There is an urgent need for people using fire in this activity to understand the danger involved and to improve their knowledge and skills in order to make a safer use of fire. The low value attributed by citizens to shrublands is also of concern. More appreciation for this type of vegetation should be promoted, as reduced appreciation may result in lower attention from the population to prevent and suppress wildfires. Our study also reveals a very interesting result in regard to citizens’ views about to the law that compel landowners to reduce fuels in the wildland-urban interface. Although it is viewed by most as an effective measure, it was considered difficult to implement, which likely explains the low degree of compliance of this law. For a more realistic and economically sustainable alternative, we recommend promoting native oak forests, making use of their high fire resistance and resilience, their minimal management requirements and high ecological value, in addition to being highly appreciated by citizens, as our study have shown. Considering the huge cost of fire fighting in this region, taking advantage of the adaptations of the native vegetation to reduce fire risks is recommended as an alternative aimed at maximizing the ecological benefits while minimizing socio-economic impacts.

##  Supplemental Information

10.7717/peerj.5657/supp-1 Supplemental Information 1 Appendix A: Questionnaire

10.7717/peerj.5657/supp-2 Supplemental Information 2 Raw data Data corresponding to the answers of the respondents to the questionnaire.

## Additional Information and Declarations

### Data Availability

The following information was supplied regarding data availability: The raw data are provided in a Supplemental File.
