# Supplementary material for: Human dimensions of wildfires in NW Spain: causes, value of the burned vegetation and administrative measures
Source: PeerJ. 2018 Sep 26;6:e5657. doi: 10.7717/peerj.5657 (PMC6163030; doi:10.7717/peerj.5657)
Supplement: Supplemental Information 1 [file peerj-06-5657-s001.docx]

**Social perception on wildfire causes, value of the burned vegetation and administrative measures.**

**SUPPLEMENTARY MATERIAL** • **APPENDIX A.**

**QuestionNaire**

The questions that follows were given in Spanish or Galician (the two official languages in Galicia), according to the language preferences of the respondent. Here we provide a translation.

**Part 1: Causes**

**Question 1**. Please, arrange these wildfire causes according to their importance, giving the maximum score (4) to the cause that originates the highest number of fires and the minimum score (1) to the cause that originates the lowest number of fires:

- Accident
- Deliberate action
- Negligence
- Natural cause

Note: the order of the causes was randomly changed in each interview.

**Question 2.** Please, classify the following specific causes according to their frequency, as *very frequent, quite frequent, infrequent* and *very infrequent*.

- Fires caused by accidents.
- Fires caused for profit gaining (e.g. to create job opportunities in fire fighting brigades or restoration activities, or to force land-use changes).
- Fires caused due to conflicts (e.g. revenges).
- Fires related to ranching activities (for pasture regeneration).
- Fires related to agriculture and vegetation management (e.g. agricultural burnings and verge maintenance).
- Fires with natural causes (lighting).
- Fires caused by pyromaniacs (mentally ill people).
- Fires related to waste management (burning of rubbish outdoors).
- Fires related to forestry works.
- Fires related to recreation activities (e.g. campfires).
- Fires related to hunting activities (e.g. to facilitate hunting).

Note: the order of the causes was randomly changed in each interview.

**Part 2: Vegetation**

**Question 3.** Please, classify the following vegetation types according to their potential fire risk, as very high, high, medium or low fire risk.

- Agriculture areas
- Eucalypt plantations
- Shrublands
- Mixed forest formations
- Pine plantations
- Native oak forests

Note: the order of the vegetation types was randomly changed in each interview.

**Question 4.** Based on the value you give to the types of vegetation mentioned before, classify them according to the importance of the loss in case they are burned by a fire, as a *very important* loss, *important* or of *low importance*:

- Agriculture areas
- Eucalypt plantations
- Shrublands
- Mixed forest formations
- Pine plantations
- Native oak forests

Note: the order of the vegetation types was randomly changed in each interview.

**Part 3: Administration role and measures**

**Question 5.** Looking at the role of the administration and its involvement in the prevention and mitigation of wildfires, please select from the following statements the one with which you are more in agreement:

The Administration is doing enough in the fight against wildfires.

The Administration is doing more than enough in the fight against wildfires.

The Administration is doing less than enough in the fight against wildfires.

**Question 6.** It follows a list of possible improvements of fire fighting measures implemented by the Administration. Which measures do you think are the most important in a scale from (1) the less important to (5) the most important.

- To improve preventive measures.
- To impose harder punishment to firesetters.
- To promote environmental education.
- To reinforce surveillance, especially in areas with higher fire risk.
- To develop legislation to prevent people to get economic benefits from wildfires.
- To increase extinction efforts.
- To shorten the reaction time after a fire starts.
- To use fire-resistant species for reforestation.

Note: the order of the measures was randomly changed in each interview.

**Question 7.** The law in Galicia establishes the obligation for landowners to clear the biomass in a 50 m band around buildings located at less than 400 m from wildland (Law 3/2007 of April 9, 2007, addressing the issues of wildfire prevention and suppression, as modified by Law 7/2012 of June 28, 2012 of Galician Forestry). Please select the assertion more in agreement with your opinion:

- I consider this law effective to prevent fires but difficult to implement by landowners.
- I consider this law effective to prevent fires and easy to implement by landowners.
- I consider this law ineffective to prevent fires.

**Demographic questions:**

- Sex (male or female)
- Age
- Place of residence (urban or rural areas)
- Do you usually perform activities outdoors e.g. for work or recreation? (yes or no).
- Educational attainment (highest level of education that the individual has completed).
